# Surgical productivity did not suffer despite the states of emergency against the COVID-19 pandemic in Japan: a retrospective observational study

**DOI:** 10.1186/s12913-022-08669-w

**Published:** 2022-10-20

**Authors:** Yoshinori Nakata, Yuichi Watanabe, Akihiko Ozaki

**Affiliations:** 1 Teikyo University Medical Information and System Research Center, Tokyo, Japan; 2 Teikyo University Graduate School of Public Health, 2-11-1 Kaga, Itabashi-ku, Tokyo, 1738605 Japan; 3 Waseda University Graduate School of Economics, Tokyo, Japan; 4 Department of Surgery, Jyoban Hospital, Fukushima, Japan

**Keywords:** COVID-19, Productivity, Malmquist index

## Abstract

**Background:**

The purpose of this study is to compute surgical total factor productivity with Malmquist index, and to evaluate the effects of states of emergency against the novel coronavirus disease 2019 (COVID-19) pandemic on its productivity change. We hypothesized that the states of emergency significantly reduced surgical total factor productivity in Japan.

**Methods:**

The authors collected data from all the surgical procedures performed in Teikyo University Hospital from April 1 through September 30 in 2019–21. Non-radial and non-oriented Malmquist model under the variable returns-to-scale assumptions was employed. The decision making unit (DMU) was defined as a surgical specialty department. Inputs were defined as (1) the number of medical doctors who assisted surgery, and (2) the duration of surgical operation from skin incision to closure. The output was defined as the surgical fee for each surgery. The study period was divided into fifty-one ten- (or eleven-) day periods. We added all the inputs and outputs of the surgical procedures for each DMU during these study periods, and computed its Malmquist index, efficiency change and technical change.

**Results:**

Seven thousand nine hundred and thirty-one surgical procedures were analyzed. The overall productivity and efficiency progressed significantly both during states of emergency and during no states of emergency. Our subgroup analysis demonstrated that there were no surgical specialties that had significantly different productivity, efficiency or technical changes between states of emergency and no states of emergency.

**Conclusions:**

We demonstrated that the surgical productivity did not suffer despite the states of emergency against the COVID-19 pandemic in Japan.

## Background

To control the novel coronavirus disease 2019 (COVID-19) pandemic, the Japanese government issued its first declaration of emergency in Tokyo on April 7, 2020 [1]. The government subsequently declared three more states of emergency against the COVID-19 pandemic in Tokyo during 2021 [2]. In response to these states of emergency, healthcare resources allocation has significantly shifted; more healthcare resources have focused on measures against the COVID-19 pandemic. As a result, routine healthcare provision in areas other than the COVID-19 countermeasures became unable to fully utilize healthcare resources, and their quality and quantity were limited. For example, the number of elective surgical procedures have been significantly reduced, and the blood products for surgery became short in supply because of the voluntary self-quarantine. In addition, more healthcare professionals have also been engaged in measures to combat the COVID-19, and shortages of manpower have occurred among staff in charge of routine health services. It might be possible that the COVID-19 countermeasures and the extreme restrictions on routine health services will collapse the entire healthcare system [3]. It has not yet been quantitatively ascertained whether the states of emergency have any effects on routine health services other than the COVID-19 countermeasures. In this study, we attempt to investigate the effects of the states of emergency on the routine health services from the perspective of healthcare productivity change.

Methods of objective measurement of efficiency and productivity have made rapid advancements in the fields of economics, business administration, and engineering since 2000 [4, 5]. Data envelopment analysis (DEA) is a standard method for measuring the efficiency of organizations and individuals. It specifically calculates efficiency by drawing a production possibility curve or an isoquant curve from the measured data by applying a linear programming method and comparing the relative distances to the curves. Malmquist index (MI) serves as an advanced mode of DEA application. It represents changes in productivity over two or more time periods. These changes in productivity can be broken down into efficiency changes and technical changes, thereby allowing for the discovery of the causes of productivity changes [6].

In this study, we chose surgery as a routine health service that was not directly related to the COVID-19 countermeasures. The purpose of this study is to compute surgical total factor productivity with Malmquist index, and to evaluate the effects of states of emergency on surgical productivity change. We hypothesized that the states of emergency significantly reduced surgical total factor productivity in Japan.

## Methods

The Teikyo University Institutional Review Board approved our study. All the methods were performed in accordance with the relevant guidelines and regulations. No consent was obtained because our present study was a retrospective observational study and the need for consent was waived by the Institutional Review Board.

### Data

Teikyo University Hospital is located in metropolitan Tokyo, Japan, serving a population of ~ 1,000,000. It has 1,152 beds and has a surgical volume of approximately 9,000 cases annually. It has thirteen surgical specialties. Teikyo University Hospital is one of the special functioning hospitals in the initial seven prefectures affected by the emergency declaration, and represents a large-scale acute care hospital profoundly impacted by the states of emergency. We collected data from all the surgical procedures performed in the main operating rooms of Teikyo University Hospital from April 1 through September 30 in 2020 and 2021. We also collected those data from April 1 through September 30 in 2019 as a control period that was before the COVID-19 pandemic. Because of our research budget and time constraints, we collected data only for six months (April-September) each year. We extracted the necessary information from surgical records in the Teikyo University Hospital electronic medical record system.

Exclusion criteria for surgery were as follows. First, surgical procedures performed under local anesthesia by surgeons were excluded. Oral, ophthalmologic, and dermatologic surgical procedures were excluded because most of their cases were minor surgeries performed under local anesthesia without anesthesiologists’ involvement, and those under general anesthesia do not represent the typical clinical activity. Second, the surgical procedures were excluded if the patients die within one month after surgery to maintain a constant quality outcome of surgery. Third, the surgical procedures that were not reimbursed under the surgical payment system in 2019–21 were excluded. Fourth, the surgical procedures were excluded if their records were incomplete for any reason.

### Malmquist index

Malmquist index (MI) represents productivity change of a decision making unit (DMU) between two time periods under dynamic situation, and is an example of comparative statics analysis [4, 5]. It is based on data envelopment analysis (DEA), which evaluates relative efficiency of DMUs against the efficient frontier under static conditions in a single period. By comparing DEA results between two time periods, MI can divide productivity change into two components, one measuring efficiency change (EC) and the other measuring technical change (TC) [6]. MI is mathematically defined as the product of EC and TC terms. The EC term relates to the degree to which a DMU improves or worsens its efficiency, while the TC term reflects the change in the efficient frontiers between the two time periods. If productivity change of a DMU is compared between Period 1 and Period 2, they are mathematically defined as follows [5],

$$\mathrm{EC}=\frac{\mathrm{Efficiency}\;\mathrm{of}\;\mathrm a\;\mathrm{DMU}\;\mathrm{in}\;\mathrm{Period}\;2\;\mathrm{with}\;\mathrm{respect}\;\mathrm{to}\;\mathrm{Period}\;2\;\mathrm{frontier}}{\mathrm{Efficiency}\;\mathrm{of}\;\mathrm a\;\mathrm{DMU}\;\mathrm{in}\;\mathrm{Period}\;1\;\mathrm{with}\;\mathrm{respect}\;\mathrm{to}\;\mathrm{Period}\;1\;\mathrm{frontier}}$$

$$\mathrm{TC}=\left(\frac{\mathrm{Efficiency}\;\mathrm{of}\;\mathrm a\;\mathrm{DMU}\;\mathrm{in}\;\mathrm{Period}\;1\;\mathrm{with}\;\mathrm{respect}\;\mathrm{to}\;\mathrm{Period}\;1\;\mathrm{frontier}}{\mathrm{Efficiency}\;\mathrm{of}\;\mathrm a\;\mathrm{DMU}\;\mathrm{in}\;\mathrm{Period}\;1\;\mathrm{with}\;\mathrm{respect}\;\mathrm{to}\;\mathrm{Period}\;2\;\mathrm{frontier}}\times\frac{\mathrm{Efficiency}\;\mathrm{of}\;\mathrm a\;\mathrm{DMU}\;\mathrm{in}\;\mathrm{Period}\;2\;\mathrm{with}\;\mathrm{respect}\;\mathrm{to}\;\mathrm{Period}\;1\;\mathrm{frontier}}{\mathrm{Efficiency}\;\mathrm{of}\;\mathrm a\;\mathrm{DMU}\;\mathrm{in}\;\mathrm{Period}\;2\;\mathrm{with}\;\mathrm{respect}\;\mathrm{to}\;\mathrm{Period}\;2\;\mathrm{frontier}}\right)^\frac12$$

$$\mathrm{MI}=\mathrm{EC}\times \mathrm{TC}$$

The MI models have been used to assess productivity change in a variety of sectors such as agriculture, airlines, banking, electric utilities, insurance companies, public sectors and healthcare [5, 6].

### Analysis framework

We employed non-radial and non-oriented Malmquist model under the variable returns-to-scale assumptions, which was particularly relevant because of its ability to employ multiple inputs and outputs simultaneously [7]. We defined the DMU as a surgical specialty department. Inputs were defined as (1) the number of medical doctors who assisted surgery (assistants), and (2) the duration of surgical operation from skin incision to skin closure (surgical duration). The output was defined as the surgical fee for each surgery. It is classified as K000- K915 in the Japanese surgical fee schedule and is called “K codes.” Each surgical procedure is assigned to one of the K codes which correspond with surgical fees. The fee is identical regardless of who (an experienced surgeon or a surgical trainee) performs surgery as long as they have medical licensure, how many assistants they use, or how long it takes to complete surgery [8, 9]. Other fees for blood transfusion, medications, special insurance medical materials and anesthesia were excluded. The monetary values of surgical fees were originally expressed in the Japanese yen and were converted to U.S. dollars at $1 = 110 yen to facilitate understanding by international readers.

### Comparison

We divided a month of the study period into three ten- or eleven-day periods; 1^st^-10^th^, 11^th^ -20^th^, and 21^st^-31^st^ (or 30^th^). We added all the inputs and outputs of the surgical procedures for each DMU during these ten- (or eleven-) day study periods, and computed Malmquist index (MI), efficiency change (EC) and technical change (TC) of each surgical specialty department using DEA-Solver-Pro Software Version 12.1 (Saitech, Inc., Tokyo, Japan) [4]. All the surgical departments in the sample were given an MI, EC and TC for each ten- (or eleven-) day period [10]. In order to more easily interpret these results, we took the natural logarithms of the MI, EC and TC, which allows us to interpret them as percent changes [11]. The natural logarithm of MI > 0 indicates progress in productivity of the DMU from Period 1 to 2, while that of MI = 0 and MI < 0 respectively indicate the status quo and deterioration in the productivity. Similarly, a natural logarithm for EC and TC measure of greater than 0 implies that there is efficiency progress and technical progress, respectively. The natural logarithm of MI equals the sum of natural logarithm of TC and that of EC [11].

We computed chronological changes of productivity, efficiency and technique in the corresponding ten- (or eleven-) day periods between 2019 and 2020, and between 2019 and 2021. There were no states of emergency against the Covid-19 pandemic in April-September 2019 in Japan. Therefore, the data in 2019 can serve as a baseline. We computed each surgical specialty’s natural logarithms of MIs, ECs, and TCs for these two intervals. We excluded early May (May 1^st^-10^th^) from our analysis because we have several national holidays during this period (so called “golden week”) every year in Japan, and surgical activity was especially low.

The following ten surgical specialty departments were included in our analysis; cardiovascular surgery, emergency surgery, general surgery, neurosurgery, obstetrics & gynecology, orthopedics, otolaryngology, plastic surgery, thoracic surgery, and urology.

### States of emergency

The states of emergency in Japan are different from city lockdowns or stay-at-home orders in other countries. The Japanese government relied on voluntary compliance. The government encouraged citizens to refrain from non-essential travel, and asked them to stay at home. Bars and nightclubs were strongly requested to close at 9 pm. However, the compliance was voluntary, not mandatory.

As of this writing in early 2022, the Japanese government has declared four states of emergency against the COVID-19 pandemic in Tokyo where Teikyo University Hospital is located. Out of these four states of emergency, the following three were within our study period; 1) April 7^th^-May 25^th^ in 2020, 2) April 25^th^-June 20^th^ in 2021, and 3) July 21^st^-Sepetember 30^th^ in 2021. The second state of emergency, which was from January 8^th^ through March 21^st^ in 2021, was out of our study period, and was not analyzed in the present study. If any day during the ten- (or eleven-) day periods are under the state of emergency, we defined that those entire ten- (or eleven-) day periods were under the state of emergency. Therefore, after excluding early May, we have seventeen periods in each year, which adds to fifty-one periods in total. Of these fifty-one periods, seventeen periods were under the state of emergency.

### Statistical analysis

We used Excel Statistics Software (SSRI Co., Ltd., Tokyo, Japan) for our statistical analysis. We compared the natural logarithms of MIs, ECs and TCs between states of emergency and no states of emergency, and those of each period against 0 using the Student t-tests. A *p*-value < 0.05 was considered statistically significant [12].

## Results

We analyzed 7931 surgical procedures performed from April 1 through September 30 in 2019–21. The characteristics of surgery are shown in Table 1. The average number of medical doctors who assisted surgery was 2.3 per case. The mean surgical duration was 145 min per case, and the mean surgical fee per surgery was $3686.

**Table 1:** Characteristics of surgery 2019–2021

Specialty	Cases	Assistants/case	Surgical duration/case(min)	Fee/case (US dollars)
**Cardiovascular surgery**	751	3.29	173	6840
**Emergency surgery**	1128	2.15	125	2840
**General surgery**	1507	2.37	190	3744
**Neurosurgery**	391	2.02	180	7545
**Obstetrics & gynecology**	1006	1.96	113	2684
**Orthopedics**	1297	2.66	127	3125
**Otolaryngology**	418	1.46	117	2104
**Plastic surgery**	322	1.98	133	2311
**Thoracic surgery**	315	2.85	175	5419
**Urology**	796	1.57	118	2790
**All surgical procedures**	7931	2.28	145	3686

Assistants/case, Surgical duration/case and Fee/case are expressed in means

The natural logarithms of MIs (percent productivity changes) were shown in Table 2. The overall productivity progressed significantly both during states of emergency (*p* = 0.025) and during no states of emergency (*p* = 0.004). It progressed by 17.2% and by 19.3% on average during states of emergency and during no states of emergency, respectively. However, there was no statistically significant difference between states of emergency and no states of emergency. The subgroup analysis demonstrated that there were no surgical specialties that had different productivity changes between states of emergency and no states of emergency.

**Table 2:** Percent productivity changes

Specialty	States of Emergency	No States of Emergency
**Cardiovascular surgery**	-4.0 ± 13.0	-2.8 ± 13.3
**Emergency surgery**	-11.4 ± 7.1	-19.8 ± 11.4
**General surgery**	-7.3 ± 7.2	-10.9 ± 16.5
**Neurosurgery**	+ 17.7 ± 11.8	+ 2.5 ± 13.9
**Obstetrics & gynecology**	-15.3 ± 4.9*	-5.9 ± 5.1
**Orthopedics**	+ 8.5 ± 16.2	-4.4 ± 7.7
**Otolaryngology**	+ 35.9 ± 18.3	+ 55.2 ± 15.9*
**Plastic surgery**	-29.6 ± 33.5	-8.8 ± 33.4
**Thoracic surgery**	+ 19.7 ± 12.3	+ 16.3 ± 8.8
**Urology**	-21.4 ± 8.8*	-35.3 ± 6.6*
**All surgical procedures**	+ 17.2 ± 7.0*	+ 19.3 ± 5.7*

The values are expressed as mean ± SE

There was no statistically significant difference between “States of Emergency” and “No States of Emergency.”

^*^Indicates that the value is significantly different from 0 (*p* < 0.05)

The natural logarithms of ECs (percent efficiency changes) were shown in Table 3. The overall efficiency progressed significantly both during states of emergency (*p* = 0.008) and during no states of emergency (*p* = 0.007). It progressed by 18.1% and by 21.2% on average during states of emergency and during no states of emergency, respectively. However, there was no statistically significant difference between states of emergency and no states of emergency. The subgroup analysis demonstrated that there were no surgical specialties that had different efficiency changes between states of emergency and no states of emergency.

**Table 3:** Percent efficiency changes

Specialty	States of Emergency	No States of Emergency
**Cardiovascular surgery**	+ 8.3 ± 17.0	+ 6.2 ± 15.7
**Emergency surgery**	+ 5.1 ± 12.7	-14.8 ± 12.5
**General surgery**	+ 16.9 ± 19.7	+ 18.9 ± 16.7
**Neurosurgery**	+ 13.7 ± 12.9	+ 3.6 ± 12.5
**Obstetrics & gynecology**	-5.6 ± 12.5	-8.2 ± 9.6
**Orthopedics**	+ 20.5 ± 15.3	+ 8.7 ± 15.9
**Otolaryngology**	+ 14.5 ± 19.6	+ 45.9 ± 22.4
**Plastic surgery**	-78.0 ± 26.4*	-53.0 ± 29.7
**Thoracic surgery**	+ 6.5 ± 14.6	-11.5 ± 12.3
**Urology**	-19.3 ± 14.0	-46.6 ± 11.7*
**All surgical procedures**	+ 18.1 ± 6.0*	+ 21.2 ± 6.8*

The values are expressed as mean ± SE

There was no statistically significant difference between “States of Emergency” and “No States of Emergency.”

^*^Indicates that the value is significantly different from 0 (*p* < 0.05)

The natural logarithms of TCs (percent technical changes) were shown in Table 4. The overall technical change was not significantly different from zero, which means that there was no technical change either during states of emergency or during no states of emergency. There was no statistically significant difference between states of emergency and no states of emergency. The subgroup analysis demonstrated that there were no surgical specialties that had different technical changes between states of emergency and no states of emergency.

**Table 4:** Percent technical changes

Specialty	States of Emergency	No States of Emergency
**Cardiovascular surgery**	-12.3 ± 10.8	-9.0 ± 9.1
**Emergency surgery**	-16.4 ± 11.1	-5.0 ± 9.2
**General surgery**	-24.2 ± 16.6	-29.8 ± 11.6*
**Neurosurgery**	+ 4.0 ± 6.2	-1.6 ± 4.5
**Obstetrics & gynecology**	-5.1 ± 11.6	+ 2.5 ± 10.1
**Orthopedics**	-10.4 ± 11.0	-13.4 ± 12.4
**Otolaryngology**	+ 21.4 ± 15.0	+ 9.4 ± 16.9
**Plastic surgery**	+ 48.5 ± 13.5*	+ 44.1 ± 10.0*
**Thoracic surgery**	+ 13.2 ± 9.2	+ 27.8 ± 10.3*
**Urology**	-5.2 ± 11.0	+ 11.3 ± 10.0
**All surgical procedures**	+ 9.5 ± 8.7	+ 5.4 ± 8.0

The values are expressed as mean ± SE

There was no statistically significant difference between “States of Emergency” and “No States of Emergency.”

^*^Indicates that the value is significantly different from 0 (*p* < 0.05)

## Discussion

We demonstrated that the surgical productivity did not suffer despite the states of emergency against the COVID-19 pandemic in Japan. On the contrary to our hypothesis presented in Background, the overall productivity progressed significantly both during states of emergency and during no states of emergency. This productivity progress was due to the positive efficiency change, not due to technical change. In addition, our subgroup analysis demonstrated that there were no surgical specialties that had different productivity, efficiency or technical changes between states of emergency and no states of emergency.

The reason for the changes above is difficult to specify from the present study because a number of public and hospital policies have changed during the pandemic. The first state of emergency started in April and ended in May 2020. In response to the government policy, Teikyo University Hospital reduced the number of elective surgeries by 20% on April 6, and by 50% on April 13. It eased the restriction to 70% on May 11, and removed all the restriction on May 25, 2020. It did not restrict the number of elective surgeries during the following two states of emergency in 2021. The restriction lasted only for approximately 50 days during the 18 months of our study period and appeared to have minimal effects on surgical productivity.

The postoperative intensive care units (ICU) were full of COVID-19 patients during the states of emergency, and major surgery was postponed during these periods. This change may have contributed to the progress in productivity and efficiency because the number of difficult surgical procedures must have reduced due to the unavailability of ICU beds. However, we have no quantitative data on the availability of ICU beds to support this speculation. We only have anecdotal episodes from our daily clinical experience.

Another possible reason for these changes might be extensive efforts of health professionals during states of emergency. The productivity progress was due to the positive efficiency change, which mostly depended on their efforts. They strived to advance the productivity of routine health services while they simultaneously treated COVID-19 patients. In addition, Tokyo did not undergo a typical COVID-19 lockdown, and it may also explain our results.

There were several surgical specialties that showed significant changes in productivity, efficiency and technique during the study periods. For example, obstetrics & gynecology and urology significantly reduced productivity during the states of emergency. It is impossible to identify any causes for these changes. There was a wide variety in MI, EC and TC among surgical specialties as evidenced by their large standard errors of means. However, no surgical specialties had significantly different productivity, efficiency or technical changes between states of emergency and no states of emergency.

There are some limitations in our study. First, the nation-wide fee schedule was revised on April 1, 2020 [8, 9]. We could not exclude the effect of this revision from our analysis in productivity change. However, our previous study demonstrated that the effects of revision was insignificant in productivity change [13]. We can assume that the effect of the revision was minimal and did not influence our conclusions. Second, this is a study conducted in a single large teaching hospital in Tokyo, Japan. Our surgical specialty departments may not represent all the departments in Japan. However, there is an advantage to studying surgical productivity in a single hospital. Since one of the significant resource inputs is ancillary services such as operating room nursing practices and availability of support personnel, all these factors are held constant in a single hospital. By comparing surgical specialty departments in the same institution, they all face the same systemic advantages and disadvantages of ancillary services [14]. Third, we had no data on the manpower availability or change in manpower during the study periods although the number of junior and senior staff in the surgical departments may be an important factor to determine their surgical productivity. Fourth, we had no data on patients’ characteristics and surgical complication rates other than the death within one month after surgery. The patient disease profile may be different during states of emergency if they fear of coming to our hospital. This may have affected the outcomes of surgical productivity. Fifth, there were a number of exclusion criteria to measure the surgical productivity. It may be possible that this selection bias may have had a significant impact on the outcomes of our study.

## Conclusions

We demonstrated that the surgical productivity did not suffer despite the states of emergency against the COVID-19 pandemic in Japan. The overall productivity and efficiency progressed significantly both during states of emergency and during no states of emergency.

## Data Availability

The data that support the findings of this study are available from the corresponding author upon reasonable requests.
